# Feedback from an avatar facilitates risk-taking by modulating the amygdala response to feedback uncertainty

**DOI:** 10.1371/journal.pbio.3003122

**Published:** 2025-04-22

**Authors:** Toshiko Tanaka, Masahiko Haruno

**Affiliations:** 1 Center for Information and Neural Networks (CiNet), National Institute of Information and Communications Technology (NICT), Suita, Japan; 2 Graduate School of Frontier Biosciences, Osaka University, Suita, Japan; Oxford University, UNITED KINGDOM OF GREAT BRITAIN AND NORTHERN IRELAND

## Abstract

With the rise of cyberspace technologies, communication through avatars has become increasingly common. However, the cognitive and neural mechanisms underlying behavioral changes induced by avatar interactions remain poorly understood, particularly when avatars serve as communication partners. To address this gap and uncover the biological mechanisms involved, we conducted behavioral (*n* = 28) and functional magnetic resonance imaging (fMRI) (*n* = 51) experiments using a simple gambling task. Participants received dynamic facial-expression feedback from either a human observer presented as an avatar or a real human face based on the outcome (win or no-win) of each gambling trial. Our results showed that expecting avatar feedback significantly increased gambling behavior in both behavioral and fMRI settings. Computational modeling revealed that differences in risk-taking behavior between the avatar and human conditions were associated with differential valuation of feedback uncertainty. Furthermore, we found that the amygdala encodes the differential valuation of feedback uncertainty, where a negative response to feedback uncertainty played a key role in choosing a gambling option. Additionally, we found that individual differences in behavioral and neural valuation of feedback uncertainty correlate with the questionnaire score that measures emotional consideration of another person’s internal states. These results demonstrate the facilitation of risk-taking behavior by avatar feedback and its underlying cognitive and neural mechanisms, thus providing deeper biological insights into risk-taking behavior and implications for human social interactions using avatars.

## Abstract 2

How does our brain process interactions with an avatar compared to real human being? This study shows that feedback from an avatar increases risk-taking behavior in humans and is associated with lower activity in the amygdala.

## Introduction

The advent of cyber technologies and the metaverse has profoundly transformed social interactions. In virtual-reality (VR) environments, individuals can adopt various forms of avatars, projecting their behaviors into a virtual realm where their interaction partners also appear as avatars. With this shift in communication style, it is essential to understand how the use of avatars influences our behavior and brain functions.

The behavioral effects of using avatars for oneself have been extensively investigated. A key example is the Proteus effect, in which an individual’s behavior in VR is shaped by the characteristics of their avatar [1,2]. Prior research has shown that using an Einstein-like avatar can enhance task accuracy requiring inspiration [3], while adopting a casual avatar can improve drumming ability [4]. Furthermore, using a “self” avatar has been linked to positive behavioral changes, such as increased proactive behavior driven by enhanced self-esteem [5]. On a neural level, studies indicate that the degree of self-body ownership in VR is negatively correlated with activity in the dorsolateral prefrontal cortex [6].

In contrast, even fewer studies have focused on behavioral changes caused by the communication partner’s avatar. In VR exposure therapy, interacting with VR avatars controlled by a human operator has been shown to reduce certain phobias and anxiety related to public speaking [7,8] or social interactions [9]. These findings suggest that avatar-based communication in VR can, to some extent, substitute for real-world human interactions. Other studies have explored how modifying an interaction partner’s behavior through an avatar (hybrid agent) impacts social interactions, yielding mixed results. While a smile-enhanced avatar increased positive emotions in social interactions [10], artificially introducing mimicry to the partner’s avatar did not significantly affect communication outcomes [11]. Thus, scientific understanding of how an interaction partner’s avatar influences behavior remains limited.

Meanwhile, companies have begun leveraging avatar-based communication in commercial settings, particularly in marketing and customer service (e.g., https://avita.co.jp/avacom/case/7C0vr9m3, https://jp.cyberlink.com/faceme/insights/cases/302/ntt-data-unstaffed-store-faceme-facial-recognition). For example, on the AVACOM insurance selection platform (https://avita.co.jp/avacom/case/7C0vr9m3), customers can choose to interact with either an avatar or real human when inquiring about insurance products. Reports from companies suggest that avatar use has doubled the number of consultation appointments, possibly because customers feel more comfortable asking questions without concerns about feedback from a real human.

Given the gap between the current scientific understanding of avatars and growing commercial applications of avatar-based communication, there is a need for a systematic investigation of how an interaction partner’s avatar affects behavior and brain function, particularly in risk-taking scenarios. To address this, we designed a task to examine how risk-taking behaviors and underlying neural computations change when a human communication partner’s appearance alternates between an avatar and a real person.

We conducted model-based behavioral and functional magnetic resonance imaging (fMRI) experiments using a gambling task to examine how human participants’ behavioral and neural responses differ depending on whether feedback comes from an avatar or a real person. We hypothesized that variations in valuation of partner feedback lead to changes in risk-taking behavior when feedback is presented through an avatar.

To analyze the behavioral and fMRI data, we constructed computational models of gambling behavior. Additionally, we collected self-report measures related to social interactions, including social anxiety (Liebowitz Social Anxiety Scale; LSAS) [12], interpersonal reactivity (Interpersonal Reactivity Index; IRI) [13], and general anxiety (State-Trait Anxiety Inventory; STAI) [14], to explore potential factors underlying individual differences in the effect of avatar use.

## Results

**Fig 1:**
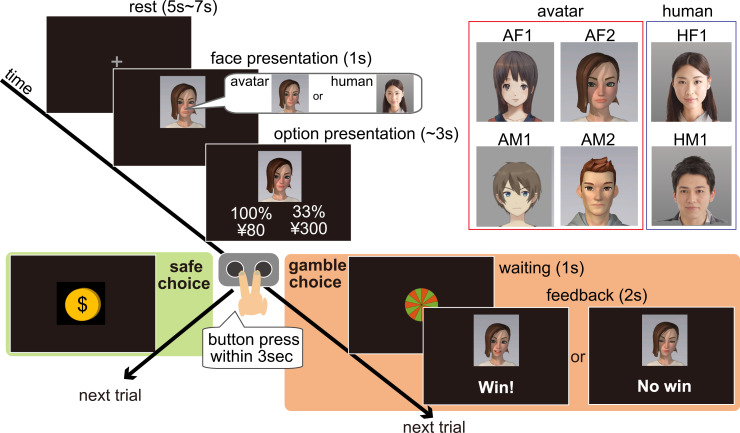
Schematic timeline of the gambling task. Participants performed a gambling task in the presence of a human observer, choosing between a safe (certain) option and a gamble (probabilistic) option. Both the participant and the observer were informed that the observer would evaluate multiple dimensions of the participant’s gambling behavior via a camera and fill in a predefined form and that the evaluation results would not be revealed to the participants. Participants were instructed to look at the observer’s face, which was displayed either as an avatar or a real human and were told that the avatar’s facial expressions and movements mirrored those of the real human observer. Participants were not given any specific guidance regarding the role of the observer’s facial expression in the task. At the start of each trial, a 4-s video of the observer’s face (unbeknownst to participants, this was a pre-recorded video) was presented before the choice phase. If the safe option was chosen, a coin image appeared, and the participant received the reward presented in the option. If the gamble option was chosen, the participant received the outcome and then viewed the observer’s facial expression feedback (a 2-s pre-recorded video displaying either a positive or negative expression). Four different avatars were used (two female avatars: AF1, AF2; and two male avatars: AM1, AM2) along with two real human observers (HF1, HM1). For privacy considerations, we replaced pictures of real human observers (HF1, HM1 in the top-right human panel) with AI-generated images which were created by using the AI Human Images (Beta) (https://www.photo-ac.com/main/genface). Each participant was paired with a same-sex human observer, whom they met in person before the experiment began.

We first conducted a behavioral experiment of the gambling task with a human observer, in which participants were asked to choose a safe or gamble option in each trial, with the safe option reward, the probability of a win (gamble option), and the expected value ratio varied (see Fig 1 and Materials and Methods). After completing the behavioral experiment, we ran an fMRI experiment using almost the same task (see Materials and Methods). Since we needed a longer inter-trial interval and repeated presentations of the same condition for the fMRI, we focused on conditions that induced different gambling rates in the avatar and human conditions in the behavioral experiment (see “Task” section in Materials and Methods). Participants of the two experiments did not overlap.

### Avatar observers promote risk-taking choices in the behavioral experiment

We first analyzed data from the behavioral experiment using an analysis of variance (ANOVA; MATLAB function: anovan) to examine whether the observer’s appearance, the reward for the safe option, and the difference in expected values influenced participants’ gambling choices. We found significant main effects for both the reward for the safe option (*F*(1,4,5,4631) = 5.01, *p* = 5.0 × 10^−^⁴) and the expected value difference (*F*(1,4,5,4631) = 6.91, *p* = 1.9 × 10^−^⁶).

Crucially, we also found a significant three-way interaction among the observer’s appearance, the reward amount for the safe option, and the expected value difference (*F*(1,4,5,4631) = 2.86, *p* = 3.3 × 10^−5^). This result indicates that the effect of the observer’s appearance on gambling choices depended on the reward amount for the safe option and the expected value difference.

**Fig 2:**
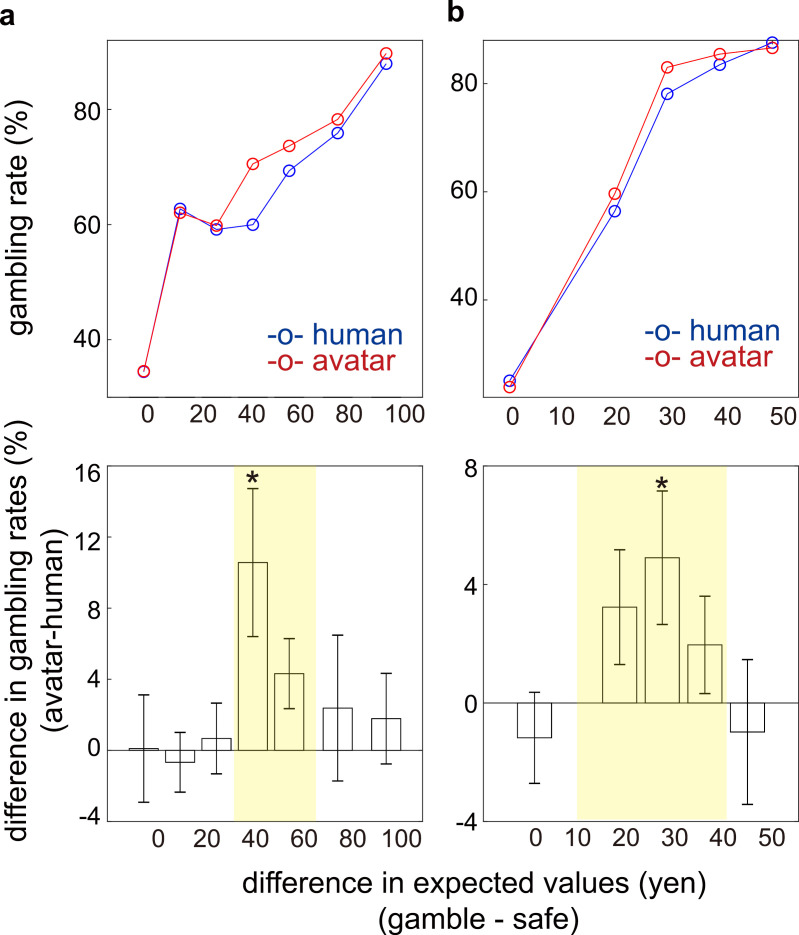
The avatar condition increased gambling choices compared with the human condition. Higher gambling rates were observed in the avatar condition in both the behavioral **(a)** and fMRI **(b)** experiments. The top panels plot the average gambling rates against the expected value differences between the gamble and safe options for the behavioral **(a)** and fMRI **(b)** experiments (red: avatar condition, blue: human condition). The bottom panels show the inter-individual averages of the intra-individual gambling rate differences (avatar–human). Error bars represent the standard error of the means. A *t* test comparing gambling rates between the avatar and human conditions showed significant differences, with *p*-values of 0.012 and 7.9 × 10^−3^, respectively. The most significant *p*-values for each panel are indicated by asterisks: *p* = 0.015 **(a)** and *p* = 8.7 × 10^−^³ **(b)**. Yellow shaded boxes highlight additional significant *p*-values: *p* = 2.3 × 10^−^³ **(a)** and 1.1 × 10^−^³ **(b)**. Numerical data are provided in S1 Data **(a)** and S2 Data **(a)**.

In Fig 2a, the top panels show the inter-individual average gambling rates as a function of the observer’s appearance and the expected value difference. The bottom panels present the inter-individual averages of the intra-individual gambling rate difference between the avatar and human conditions. These data confirm that gambling rates were significantly higher in the avatar condition than in the human condition, particularly when the expected value difference was intermediate.

### Higher gambling rates in the avatar condition were replicated in the fMRI experiment

Next, we analyzed data from the fMRI experiment using the same ANOVA model as in the behavioral experiment to examine the effects of the observer’s appearance, the safe option reward, and the expected value difference on gambling choices.

As shown in Fig 2b, we found significant main effects for the reward amount for the safe option (*F*(1,3,4,5238)=13.35, *p*=1.1 × 10^−8^) and the expected value difference (*F*(1,3,4,5238) = 72.48, *p* = 7.5 × 10^−60^). Crucially, the three-way interaction was also significant (*F*(1,3,4,5238) = 4.03, *p* = 2.0 × 10^−4^), further supporting that the avatar condition promoted risk-taking choices, particularly at intermediate expected value differences, in both experiments.

We also tested the effect of gender by conducting an ANOVA using a model that replaced the observer’s appearance with the participant’s gender, since previous studies have reported gender differences in responses to social stimuli, including facial expressions [15]. However, the three-way interaction was not significant in either experiment (behavioral experiment: *F*(1,4,5,4631) = 0.99, *p* = 0.44; fMRI experiment: *F*(1,3,4,5238) = 1.68, *p* = 0.15). Similarly, the interaction between gender and the observer’s appearance was not significant (behavioral experiment: *F*(1,1,4631) = 0.030, *p* = 0.86; fMRI experiment: *F*(1,1,5238) = 0.38, *p* = 0.54). Given these findings, we conducted the subsequent analysis without including gender as a factor.

### Model selection

To identify the mechanism underlying the higher rate of risk-taking in the avatar condition, we constructed and compared computational models. We hypothesized that valuation of feedback uncertainty for facial-expression feedback influences the differential gambling rate in the avatar and human conditions and therefore included it in the full (value) model. We also incorporated all other factors in the full model that can influence gambling rates: subjective probability weighting [16–17] for expected reward, loss aversion [18], variance risk of reward [19], and the effect of previous outcomes [20] (see “Computational models” in the Materials and Methods section, Eq 10 and S1 Table #50).

Next, we assessed each term in the full model by removing a term one by one and identified the best models using the Akaike’s information criterion (AIC) [21] and Bayesian information criterion (BIC) [22]. As a result, we found that the best model consists of the subjective gambling and safe-option rewards term and the valuation of feedback uncertainty term and that the coefficient for the feedback uncertainty should be estimated separately for the avatar and human conditions (16−6 in S2 Table; see Eq 13 in Materials and Methods).

### Valuation of feedback uncertainty is pivotal for differential gambling behavior

**Fig 3:**
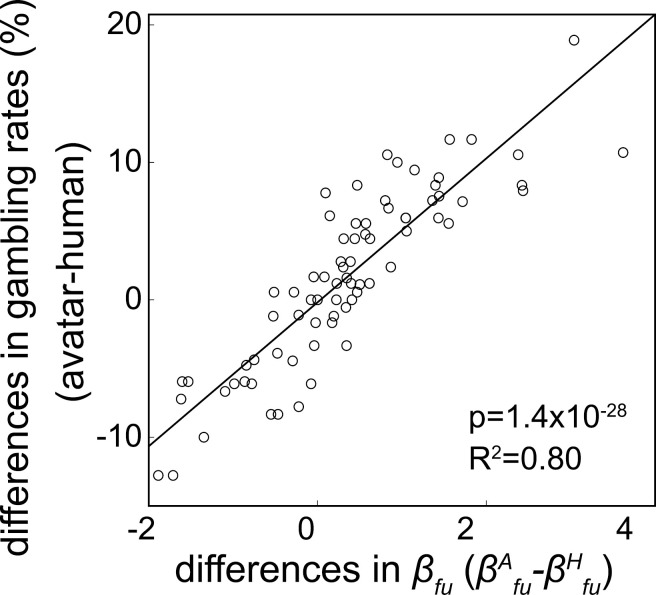
Difference in gambling rates correlated with differential feedback uncertainty coefficient. $\beta_{fu}^{A}-\beta_{fu}^{H}$. A significant correlation was observed between the differential average gambling rate (%) and the differential feedback uncertainty coefficient (i.e., $\beta_{fu}^{A}-\beta_{fu}^{H}$). Both behavioral and fMRI samples are included in the dot plot (*p* = 1.4 × 10^−28^, *R*² = 0.80). Each point represents a single participant. Numerical data are provided in S1 Data **(a, b)** and S2 Data **(a, b)**.

The result of the previous section suggests that differences in the feedback uncertainty coefficients (valuations) $(\beta_{fu}^{A}-\beta_{fu}^{H})$ between the avatar and human conditions may explain differences in the gambling rates. Indeed, as shown in Fig 3, we found a significant correlation between differential coefficients $(\beta_{fu}^{A}-\beta_{fu}^{H}$, x-axis) and differences in gambling rates (y-axis) (*p* = 1.4 × 10^−28^, *R*^2^ = 0.80). We also confirmed that $\beta_{fu}^{A}$ in the avatar condition was larger than $\beta_{fu}^{H}$ in the human condition (S1 Fig.; *p* = 0.0021, *t* = 2.96). These results demonstrate that the differential valuation of feedback uncertainty (i.e., $\beta_{fu}^{A}-\beta_{fu}^{H}$) plays a key role in producing different gambling rates in the two conditions.

### Increased risk-taking in the avatar condition correlated with consideration of the other’s state

Our model-based analysis revealed that different gambling rates across observer appearances were driven by differential valuation of feedback uncertainty. To identify the underlying cause, we examined the correlation between differential uncertainty feedback valuation and personality trait scores, including general anxiety (STAI), social anxiety (LSAS), and inter-personal reactivity/empathy (IRI).

**Fig 4:**
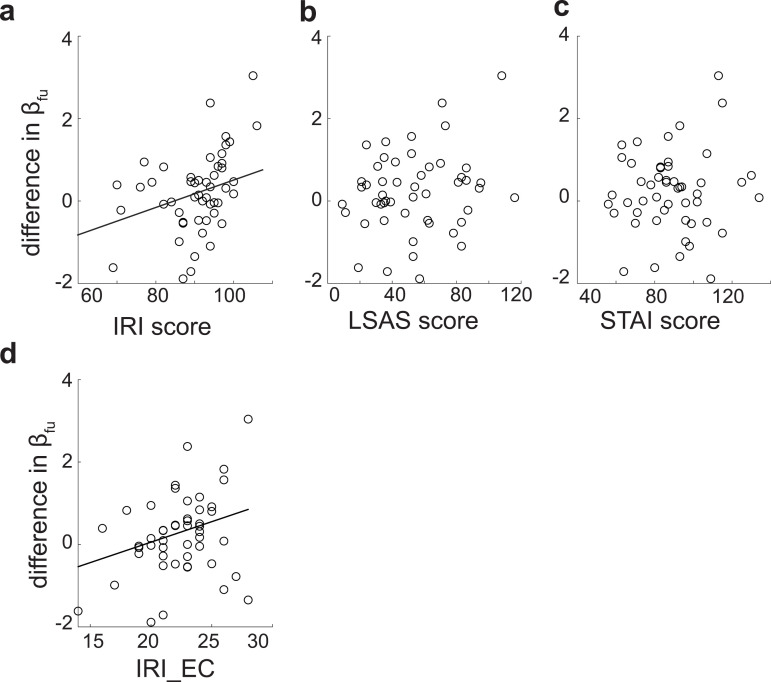
Inter-personal reactivity scores correlated with differential $\beta_{fu}$. We examined correlations between self-reported personality scores (IRI, LSAS, and STAI) and differential $\beta_{fu}$ **(a–c)**. The IRI score was significantly correlated with $\beta_{fu}$ (*p* = 0.027 and *R*^2^ = 0.17), whereas LSAS and STAI scores did not show significant correlations. **(d)** The correlation between IRI sub-scores (IRI_FA, IRI_PT, IRI_EC, and IRI_PD) and $\beta_{fu}$. Among these sub-scores, only IRI_EC showed a significant correlation (*p* = 0.019 and *R*^2^ = 0.093), as indicated by the black regression line. The other sub-scores were not significantly correlated (IRI_FA: *p* = 0.27 and *R*^2^ = 0.078, IRI_PT: *p* = 0.13 and *R*^2^ = 0.067, and IRI_PD: *p* = 0.24 and *R*^2^ = 0.088). Robust regression was used to minimize the effect of outliers, and *p*-values were corrected for multiple comparisons using the Benjamini–Hochberg method. Numerical data are provided in S2 Data **(b, c)**.

Fig 4 plots these trait scores against the differential $\beta_{fu}\left(\text{i.e}.,\beta_{fu}^{A}-\beta_{fu}^{H}\right)$. The results indicate that only IRI was significantly correlated with differential $\beta_{fu}$ (IRI: Fig 4a; *p* = 0.027 and *R*^2^ = 0.17), while no significant correlations were found for LSAS (Fig 4b; *p* = 0.60) or STAI (Fig 4c; *p* = 0.98). Furthermore, among the four IRI sub-scores, only empathetic concern (EC) showed a significant correlation with differential $\beta_{fu}$ (EC: Fig 4d; *p* = 0.019 and *R*^2^ = 0.093). These findings suggest that emotional consideration of the other’s feelings or states is the key factor associated with differential valuation of feedback uncertainty.

### Amygdala encodes differential valuation of feedback uncertainty in avatar and human conditions

Above we show that differential valuation of feedback uncertainty explains differential gambling rates in the avatar and human conditions. Therefore, we focused on differential feedback uncertainty in our fMRI analysis.

**Fig 5:**
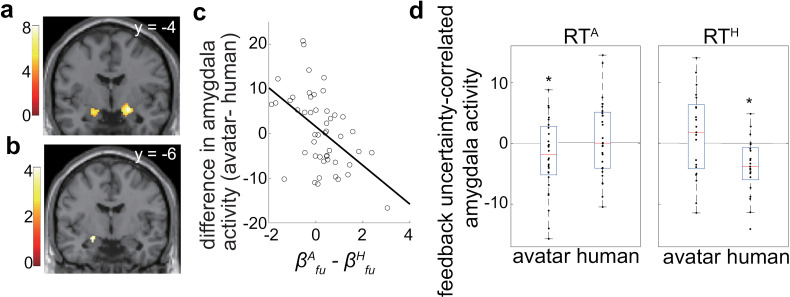
Amygdala response to feedback uncertainty explains differential risk-taking in avatar and human conditions. **(a)** Whole-brain subtraction analysis comparing avatar and human observer conditions revealed lower amygdala activity in the avatar condition. Peak activity was identified in the CM (MNI coordinates: [20 −4 −12] and P_FWE_ = 8.5 × 10^−6^, *T*-value = 8.21, *Z*-value = 6.46). For display purposes, a threshold of *p*_unc._ < 10^−4^ and *k* > 100 was applied (*y* = −4). **(b)** To find brain regions associated with differential valuation of feedback uncertainty ($\beta_{fu}$), we weighted differential activity correlated with feedback uncertainty (avatar–human) by the differential behavioral coefficient for feedback uncertainty (i.e. $\beta_{fu}^{A}-\beta_{fu}^{H}$). A significant correlation was observed in the CM, with an FWE-corrected *p*-value of 0.039 at the peak (small-volume corrected using the Anatomy toolbox CM ROI). For display purposes, a threshold of *p*_unc._ < 0.001 and *k* > 10 was applied (*y* = −6). **(c)** Activity correlated with differential feedback uncertainty at the center of an independent anatomical ROI of the CM is plotted against the differential feedback uncertainty coefficient ($\beta_{fu}^{A}-\beta_{fu}^{H}$). The negative slope indicates that participants who exhibited greater risk-taking behavior in the avatar condition had a lower feedback uncertainty-correlated response in the CM during interactions with avatars. **(d)** Participants were divided into two groups: risk-takers against avatars (RT^A^; left panel) with higher values of $\beta_{fu}$ in the avatar condition $(\beta_{fu}^{A}>\beta_{fu}^{H}),$ and risk-takers against humans (RT^H^; right panel) with lower values of $\beta_{fu}$ in the avatar condition $\left(\beta_{fu}^{H}>\beta_{fu}^{A}\right)$. CM responses to feedback uncertainty were compared with zero separately for the avatar and human conditions within each group. In RT^A^ (left), the CM response was significantly negative in the avatar condition (*p* = 4.7 ×10^−2^, *t* = −1.73), while in RT^H^ (right), the CM response was negative in the human condition (*p* = 5.6 × 10^−**4**^, *t* **=** −3.79). Asterisks indicate statistical significance. Numerical data are provided in S2 Data **(b, d)**.

We performed a general linear model (GLM) analysis of the fMRI data by setting up separate regressors for two option-presenting events for avatar and human conditions. Each event was associated with three parametric modulators: the feedback uncertainty and the expected values of two options (see “GLM analysis” section in Materials and Methods). First, we contrasted brain activity between the avatar and human conditions when deciding to choose a safe or gamble option (i.e., avatar–human) and found a significant decrease in activity only in the amygdala (*p* = 8.5 × 10^−6^, familywise error [FWE] corrected; Fig 5a). Therefore, we chose the amygdala as our primary target in the subsequent analysis and used the amygdala region defined in the anatomy toolbox (JuBrain Anatomy Toolbox/) as our region of interest (ROI).

Because we aimed to identify brain regions associated with differential valuatino of feedback uncertainty ($\beta_{fu}$), we weighted first-level differential activity correlated with feedback uncertainty (avatar–human) by the differential behavioral coefficient for feedback uncertainty (i.e., $\beta_{fu}^{A}-\beta_{fu}^{H}$) in the second level using SPM12 (see Materials and Methods). As shown in Fig 5b, we found a significant correlation in the central amygdala (CM) (with an FWE-corrected *p*-value of 0.039 at the peak). We confirmed the negative correlation between activity (beta value) for differential feedback uncertainty at the center of an independent CM anatomical ROI (left CM in the anatomy toolbox) and differential $\beta_{fu}$ ($\text{i.e.},\beta_{fu}^{A}-\beta_{fu}^{H}$) (Fig 5c). These results show that the CM contributes to differential valuatin of feedback uncertainty in the avatar and human conditions.

Next, to further investigate the link between the CM response to feedback uncertainty and risk-taking behavior, we divided participants into two groups: risk-takers against avatars (RT^A^; Fig 5d left panel), i.e., those with higher values of $\beta_{fu}$ in the avatar condition $(\beta_{fu}^{A}>\beta_{fu}^{H});$ and risk-takers against humans (RT^H^; right panel), i.e., those with lower values of $\beta_{fu}$ in the avatar condition $\left(\beta_{fu}^{H}>\beta_{fu}^{A}\right)$. We compared CM responses to feedback uncertainty with zero separately for the avatar and human conditions within each group and found that for RT^A^, the CM response was negative in the avatar condition (*p* = 4.7 × 10^−2^, *t* = −1.73), while for RT^H^, the CM was negative in the human condition (*p* = 5.6 × 10^−4^, *t* = −3.79). These observations indicate that a negative amygdala response to feedback uncertainty drives risk-taking behavior.

### CM response to feedback uncertainty in avatar condition correlates with the IRI EC score

**Fig 6:**
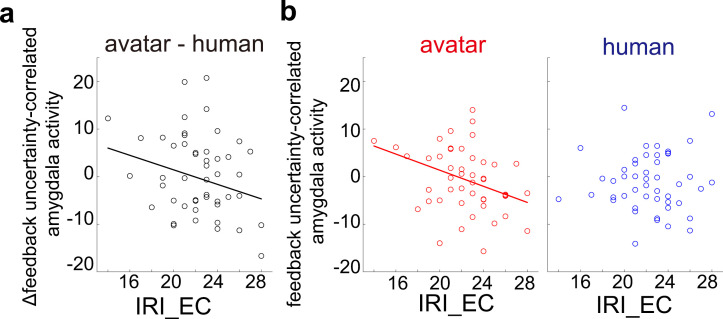
Differential response of the CM to feedback uncertainty correlates with the IRI_EC score. **(a)** The differential response of the CM to feedback uncertainty is plotted against the IRI_EC score and shows a significant correlation (*p* = 0.042, *R*^2^ = 0.071). **(b)** CM responses to feedback uncertainty are plotted against the IRI_EC score separately for the avatar and human conditions. We found a significant collation in the avatar condition (left; *p* = 5.1 × 10^−3^ and *R*^2^ = 0.098) but not in the human condition (right; *p* = 0.65, *R*^2^ = −0.0021). Regression lines indicate a significant correlation. Robust regression was used in the estimation to mitigate the effect of outliers. Numerical data are provided in S2 Data **(c, d)**.

To explore factors which control higher valuation of feedback uncertainty in the avatar condition, we examined whether IRI scores can explain individual differences in the amygdala responses to feedback uncertainty. We found a significant correlation between the differential response to feedback uncertainty and the IRI_EC score (Fig 6a; *p* = 0.042, *R*^2^ = 0.071). Importantly, we found that the CM response to feedback uncertainty in the avatar condition correlated with the IRI_EC score (Fig 6b left; *p* = 5.1 × 10^−3^ and *R*^2^ = 0.098) but not in the human condition (Fig 6b right; *p* = 0.65, *R*^2^ = −0.0021). These results suggest that a greater emotional consideration of the other’s internal states is correlated with a more negative amygdala response to feedback uncertainty in the avatar condition, leading to an increase in risk-taking behavior.

### Other brain regions encoding differential valuation of feedback uncertainty

We have so far focused on the amygdala but conducted a whole-brain fMRI analysis to identify other brain regions representing differential valuation of feedback uncertainty in the avatar and human conditions. Specifically, we conducted the same analysis as done on the amygdala and adopted a moderate statistical threshold (i.e., *p* < 0.001 uncorrected and cluster size > 100).

**Fig 7:**
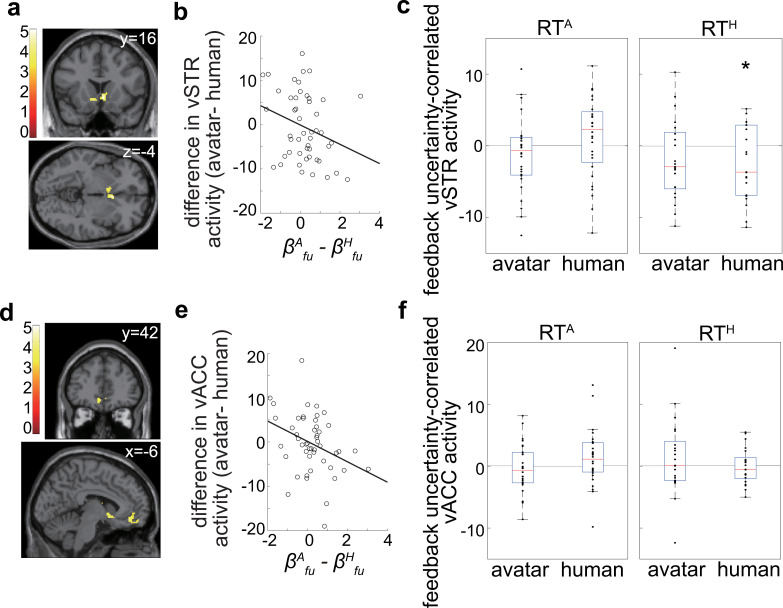
Feedback uncertainty-correlated activity in the vSTR and vACC. **(a, d)** Differential activity correlated with feedback uncertainty (avatar–human) was weighted by the differential behavioral coefficient (valuation) for feedback uncertainty (i.e. $\beta_{fu}^{A}-\beta_{fu}^{H}$). Significant activity was identified in the vSTR **(a)** (peak MNI coordinates: [6,16, −4], FWE-corrected *p* = 1.5 × 10^-5^) and the vACC **(d)** (peak MNI coordinates: [−6, 42, −12], FWE-corrected *p* = 0.030). **(b, e)** Activity correlated with differential feedback uncertainty at the center of independent anatomical ROI of the vSTR **(b)** and vACC **(e)** is plotted against the differential feedback uncertainty coefficient (i.e. $\beta_{fu}^{A}-\beta_{fu}^{H}$). **(c, f)** Responses (beta values) of the vSTR **(c)** and vACC **(f)** to feedback uncertainty are displayed separately for the avatar and human conditions within RT^A^ and RT^H^, following the same format as in Fig 5d. In the vSTR **(c)**, feedback uncertainty-correlated activity was negative in the human condition of RT^H^ (*p* = 0.024, *t* **=** −2.10) but not in the avatar condition of RT^A^ (*p* = 0.15, *t* = −1.02). No significant difference was found between the avatar and human conditions in RT^A^ (*p* = 0.075, *t* = −1.85). In the vACC **(f)**, feedback uncertainty-correlated activity showed no significant difference between the avatar and human conditions in either RT^A^ or RT^H^. The asterisk indicates statistical significance. Numerical data are provided in S2 Data **(b, d)**.

We found that the ventral striatum (vSTR) and ventral anterior cingulate cortex (vACC) exhibited responses correlated with differential feedback uncertainty weighted by differential behavioral valuation of feedback uncertainty ($\text{i.e.},\beta_{fu}^{A}-\beta_{fu}^{H}$). The vSTR showed a peak response at Montreal Neurologic Institute (MNI) coordinates [6,16, −4] with a cluster level FWE-corrected *p*-value of 1.5 × 10^−5^ (Fig 7a). Similarly, the vACC exhibited a peak response at MNI coordinates [−6, 42, −12], with a cluster level FWE-corrected *p*-value of 0.030 (Fig 7d). Thus, despite the moderate statistical threshold, both regions demonstrated robust activity.

Fig 7b and 7e illustrate that both the vSTR and vACC exhibited a negative slope between their differential response (at the center of an independent anatomical ROI) to feedback uncertainty and the differential behavioral valuation of feedback uncertainty. These results suggest that the vSTR and vACC function similarly to the amygdala, exhibiting both differential responses to feedback uncertainty and correlation with differential behavioral valuation of feedback uncertainty.

However, we also found that the vSTR and vACC encode less information about risk-takers against avatars (RT^A^) in the avatar conditions than does the amygdala. More specifically, in the vSTR, a significant negative response to feedback uncertainty was observed only in the human condition of RT^H^ (*p* = 0.024, *t*= −2.10) (Fig 7c) but not in the avatar condition of RT^A^ (*p* = 0.16, *t* = −1.02). Similarly, in the vACC, no significant difference between the avatar and human conditions was observed in either RT^A^ or RT^H^ (Fig 7f). These results indicate that the vSTR and vACC play a less critical role in driving increased risk-taking behavior in the avatar condition.

## Discussion

In this study, we investigated how facial expression feedback from an observer—whether an avatar or a real person—affects risk-taking behavior in a gambling task (Fig 1). We found that expecting feedback from avatars facilitated risk-taking behavior compared with real-human observers (Fig 2).

Our computational model-based analysis (S1 and S2 Tables) revealed that the increased gambling rate in the avatar condition was driven by a heightened valuation of feedback uncertainty (Fig 3). Furthermore, individual differences in valuation of feedback uncertainty between the avatar and human conditions correlated with a personality trait score that reflected an emotional consideration for others (i.e., the IRI_EC score) (Fig 4).

Our fMRI analysis further demonstrated that differential valuation of feedback uncertainty between the avatar and human conditions was associated with differential amygdala responses to feedback uncertainty (Fig 5). Notably, a negative amygdala response to feedback uncertainty contributed to increased risk-taking behavior in both the avatar and human conditions. Additionally, in the avatar condition, amygdala activity correlated with the IRI_EC score (Fig 6). These findings suggest that the amygdala’s response to feedback uncertainty plays a key role in driving increased risk-taking behavior in the avatar condition and that this function is closely linked to individual differences in interpersonal (emotional) reactivity.

We also observed differential responses to feedback uncertainty between the avatar and human conditions in the vSTR (Fig 7a) and vACC (Fig 7d). Unlike the amygdala, these regions did not exhibit significant differences in feedback uncertainty-related activity between the avatar and human conditions in participants who showed higher risk-taking behavior toward avatars (i.e., RT^A^; Fig 7c and 7f). Additionally, their activity was not correlated with the IRI_EC score. These results suggest that the amygdala is the primary driver of increased risk-taking behavior in the avatar condition, likely by interacting with the vSTR and vACC.

Previous studies have reported strong bidirectional projections among the amygdala, vSTR, and vACC [23–26], with some pathways implicated in reward-based decision-making and others in emotion regulation [27] and cognitive control [28]. Our fMRI results suggest that emotional signals from the amygdala influence standard risk-taking decision-making processes in the vSTR and vACC in a bottom-up manner (i.e., from emotion to cognition).

Our findings highlight the pros and cons of using avatars in the real world. On the positive side, people may communicate and make decisions better with others in a less stressful manner and take more risks, which may prevent them from missing opportunities for learning and growth. As reported by the World Values Survey, modern people are becoming increasingly conservative (World Values Survey; https://www.worldvaluessurvey.org/WVSNewsShow.jsp?ID=427), and the use of avatars may weaken this trend. Our questionnaire results suggest that this effect of avatars is stronger in those who emotionally consider others’ states in their decision-making. As a result, the use of avatars may promote economic activity, such as trading and investing. However, on the negative side, it is important to remain aware that we may unconsciously take too many risks when we communicate with avatars compared with real humans. 

Related to this, our findings may also have implications into basic human experiments since human neuroscience [29], cognitive science [30], psychology [31], and economics [32] have often used avatars in their experimental paradigms. We need to be more cautious about the influence of avatar partners in such experiments.

The findings of the present study pose a natural question of whether the increase in risk-taking behavior is exclusive to the use of avatars. We think the answer is no. The variability in the appearance of real-human observers would also likely affect risk-taking behavior through a similar mechanism to the one we report here. Regardless, because the use of avatars will become more common in the future, it would be beneficial for society to understand how the use of typical avatars can change human behavior. Furthermore, with rapid technological advances, it is now possible to create more elaborate human-like avatars. Future investigations using such avatars are important.

One possible counterargument is that avatars may have simply caused participants to ignore the feedback. However, our findings suggest that this is unlikely. First, we demonstrated that the increased risk-taking behavior was driven by a negative amygdala response to feedback uncertainty rather than by a lack of neural activity. Second, our pilot experiment (S2 Fig), which included two conditions, one in which the avatar and real-human observers were switched approximately every 10 trials and another without any observer, showed that the avatar-observer condition elicited more gambling behavior than both the real-human and no-observer conditions. Importantly, gambling rates were comparable between the real-human and no-observer conditions, further supporting our conclusions.

The amygdala plays a central role in emotion processing [33–34], and its hyperactivity is associated with negative processing bias, a key feature of various mental disorders [35]. Previous studies have also linked the amygdala to gambling behavior; for example, amygdala volume has been correlated with differences in subjective probability parameters (similar to Eq 4) between gambling-disorder patients and healthy controls [36]. Additionally, an fMRI study on social anxiety and gambling behavior in the presence of human facial-expression feedback found that individuals with higher social anxiety scores (i.e., LSAS) were more likely to choose safe options and exhibited increased amygdala activity [37]. In contrast to these studies, we demonstrated that a negative amygdala response to feedback uncertainty was the key to increasing risk-taking behavior.

Furthermore, we assessed social anxiety (LSAS) and interpersonal reactivity (IRI) and found that the effect of avatars was correlated with IRI but not with LSAS. This finding suggests that the influence of avatars is more closely related to the emotional demands of mentalizing about real humans rather than the physical avoidance of social interactions.

This study has several limitations. First, we only examined the effects of four avatars with moderate attractiveness. Future research should explore whether avatar effects vary based on their similarity to the participant or their specific characteristics. It would also be valuable to investigate whether the same effects occur when avatars closely resemble real individuals. Additionally, we only tested same-sex participant-avatar pairs to reduce potential confounds, but the effects may differ in opposite-sex interactions and should be examined in future studies. Finally, the influence of age on these effects remains an open question.

Despite these limitations, the present study revealed that the use of avatars as an interface for communicating with a partner facilitates risk-taking behavior by increasing the valuation of feedback uncertainty and that the amygdala is central to this facilitation. These findings should provide valuable insights into human communications and social interactions using avatars that are becoming increasingly more common in our world.

## Materials and methods

### Experimental procedures

#### Participants.

We first estimated the number of participants using G-power (Smith, J. 2023; Version 3.1.9.7. https://www.psychologie.hhu.de/arbeitsgruppen/allgemeine-psychologie-und-arbeitspsychologie/gpower.html). According to Lipsey and Wilson [38], the effect size in psychological experiments is generally of medium magnitude on average. We adopted this assumption, and the estimated sample size for the behavioral experiments was 27 (medium effect size; *d* = 0.5, Power = 0.8, *α* = 0.05). To evaluate the sample size for the fMRI experiments, we used our previous amygdala imaging data [39] to estimate the effect size (*f*^2^); it was slightly larger than 0.15 (about 0.2). Therefore, we set the effect size to be medium (*f*^2^ = 0.15) for the linear regression. The estimated sample size was 55 (Power = 0.8, *α* = 0.05).

Participants were recruited from the local population (mainly university students) via our online recruitment system and gave informed consent for participating in the experiments. This study adhered to the Declaration of Helsinki and was approved by the ethics committee at the Centre for Information and Neural Networks (CINET), Osaka, Japan (approval number: B210142205). Informed consent was obtained from all participants both orally and in writing.

One hundred and four healthy participants (18–27 years old with no history of mental illness) participated in the two experiments: 34 in the pilot behavioral experiment and 70 in the fMRI experiment. Among them, 25 participants were excluded from the subsequent analysis: 1 decided from the outset not to gamble, 6 did not understand the task, 2 suspected the reality of the interaction with an observer, and 16 missed button presses or reported pressing wrong buttons more than three times. As a result, data from 28 participants for the behavioral experiment (mean age, 21.7; SD, 1.98; 17 females) and 51 participants for the fMRI experiment (mean age, 21.9; SD, 1.65; 27 females) were included in the analysis.

#### Task.

To investigate the effect of using avatars on risk-taking behavior, a simple gambling task was used with either avatar or real-person facial feedback (see Fig 1). Participants were instructed to perform the task while looking at the face of the partner (observer) over the camera who received the experimental instruction together with the participant before starting the experiment. In reality, the two real human (one female and one male) observers were recruited and came to the laboratory to record a video of their dynamic facial expressions (video) in advance, and their preprepared video clips were used in the experiments.

In the instruction phase of the task, both the participant and the observer were informed that the observer would evaluate multiple dimensions of the participant’s decision-making by filling out a predefined form. They were also told that the observer would receive more detailed instructions later and that the evaluation results would not be revealed to the participant.

In each trial of the task, the observer’s neutral face movie was first displayed for 1 s, and two options were then presented below the face window (movie continued): one was the safe option with a smaller certain reward and the other was the gambling option with an uncertain (probabilistic) large reward. Participants were asked to choose one of the two options by pressing a button within 3 s. If the participant chose the safe option, he/she obtained the reward, and the task proceeded instantly to the next trial. If the probabilistic option was selected, the outcome depended on the win probability presented in the option.

At the timing of the outcome presentation of gamble choices, participants viewed facial expression feedback from the observer (we used a 2s movie unknown to the participants). That is, participants viewed positive and negative facial expressions when they won and lost (no-win), respectively. Importantly, the observer’s appearance was switched between an avatar and a real human roughly every 10 (6–13) trials.

Notably, although the participants were informed that the observer evaluated risk-taking behaviors across multiple dimensions and completed a predefined form, they were not given any specific guidance regarding the role of the observer’s facial expressions in the task. The participants were also informed that their choices had no effect on the observer’s gratuities and that the observer’s evaluation had no effect on their rewards. We hypothesized that participants change their risk-taking behavior in the avatar and human conditions as an effect of emotion. After the experiment, we confirmed that participants understood their own rewards and the observer’s rewards were independent and that they believed that they were connected with their partner online via cameras.

In the behavioral experiment, the total number of trials was 168 (84 for each observer condition), and the reward for the gambling options was 300 yen (~US $2). The duration of the rest period (inter-trial interval) varied between 1 and 2 s, and the win probability was 10, 33, and 50%. For each win probability, seven different rewards for the safe options were prepared, with the value ratio between the gamble and safe options (gamble value/safe value) ranging between one and three. We only used the AF1 and AM1 avatars (Fig 1), and participants were paired with the observer of the same sex.

In the fMRI experiment, due to time constraints and the need for repeating the same conditions, we only used the options that produced differences between the avatar and human conditions in the behavioral experiment. The fMRI experiment consisted of 108 trials (54 for each observer condition), and the probabilities used in the gambling options were 22, 33, and 44%. The value ratio between the gamble and safe options was 1, 1.25, 1.5, 1.75, and 2 for 33%, and 1 and 1.5 for the other two probabilities.

We used four avatars (AF1, AF2, AM1, and AM2; one participant experienced only one avatar; see Fig 1). Among these four avatars, we did not find a significant difference in the gambling rate (AF1-AF2, *p* = 0.099; AF1-AM1, *p* = 0.43; AF1-AM2; *p* = 0.75; AF2-AM1, *p* = 0.61; AF2-AM2, *p* = 0.84; and AM1-AM2, *p* = 0.30). Therefore, we analyzed the data for all avatars together.

Participants were interviewed for their impressions of the avatars after the experiment. The post-experiment questionnaire showed no difference in the likability of the avatars (*p* = 0.28 and 0.27, positive and negative face, respectively). Participants obtained a reward depending on what they earned during the task. Finally, they were asked to complete personality trait questionnaires online within a week after the experiment. Note that we did not use the terminologies “gamble” or “safe” in the instructions.

### Avatars and dynamic facial expressions

We used free materials in Live2D (hibiki for AM1 and izumi for AF1) and avatars crafted using free parts from FaceRig for AM2 and AF2 (see Fig 1 for avatars). Transformations of the observer’s facial expressions and movements onto an avatar were performed using FaceRig. In Fig 1, to protect privacy, we replaced real-human faces of observers we used in our experiments with AI-generated images (photoAC; https://www.photo-ac.com/).

### Personality trait questionnaire

We used the IRI as a composite measure of interpersonal characteristics. IRI is a 28-item scale consisting of the following sub scores: fantasy (FA), perspective taking (PT), EC, and personal distress (PD). Unless otherwise stated, the total score was used as the IRI score. To evaluate social anxiety, we used the LSAS. LSAS assesses both social anxiety in situations (LSAS_fear) and avoidance of those situations (LSAS_avoidance). Unless otherwise stated, we used the total score of the LSAS. To evaluate general anxiety, we used the STAI. STAI assesses both trait-anxiety and state-anxiety. Unless otherwise stated, we used the total score of the STAI. One participant who did not answer the questionnaire was excluded from the analysis of personality scores.

### Behavioral analysis

An ANOVA was first conducted to test whether factors including the observer’s appearance, the difference in the expected values between options, and the reward amount of the safe option, influenced the choices of the participants. Next, we used paired *t*-tests to further investigate the within-individual change of the gambling rate in the avatar and human conditions. The null hypothesis was that the mean difference in the gambling rate between observer conditions is zero.

### Computational models

The prospect theory [16–17] postulates that the loss aversion can alter gambling rates [18], and some participants told us in the post-experimental interview that they felt as if gambling would decrease the reward amount that they were supposed to receive. Monetary reward factors such as subjective reward sensitivity and variance risk [19] can influence gambling rates. It is also possible that the outcome of the previous trial may impact gambling rates [20]. We incorporated all these factors into our full utility (value) model consisting of the monetary term (MONEY), face-related terms (FACE), and the previousResult term as shown in Eq 1.


$$U_{gamble}=MONEY+FACE+previousResult$$ (1)

*MONEY* comprises the subjective expected reward, monetary risk represented by the variance risk, and the loss aversion terms as in Eq 2.


$$MONEY=subRew_{gamble}+monetaryRisk+LOSSAv$$ (2)

The subjective reward for the gambling option ($subRew_{gamble})$ was calculated as Eq 3 using the subjective probability (*Wp*; Kahneman and Tversky, 1979) [16] shown in Eq 4


$$subRew_{gamble}=\beta_{g}*Wp*R_{g}^{\theta_{1}}$$ (3)

Wp=pγpγ+1−pγ1γ (4)

β_g_ is the coefficient for monetary reward for gambling, and $R_{g}$ represents the reward size for gambling. pand γ represent the win probability and the subjective distortion index of the win probability.

monetaryRisk,LOSSAv, andFACE are computed as illustrated in Eqs 5–7.


$$monetaryRisk=\beta_{risk}*variance$$ (5)

$$LOSSAv=\beta_{loav}*\left(1-Wp\right)*R_{s}^{\theta_{2}}$$ (6)

$$FACE=\beta_{win}*Wp-\beta_{nowin}*\left(1-Wp\right)+\beta_{fu}*entropy$$ (7)

$\beta_{risk},\beta_{loav,}\beta_{win},\beta_{nowin}$, and $\beta_{fu}$ are the coefficients of monetary risk, loss-aversion, win, nowin, and feedback uncertainty, respectively. $R_{s}$ and $\theta_{t}$ (*t* = 1 or 2) represent the reward size for the safe option, and the subjective distortion index of the monetary reward for the safe and gamble options, respectively.

The first and second terms in Eq 7 represent the positive and negative effects of the two facial expressions, respectively, and the third term represents the facial feedback uncertainty using entropy. Since the subjective probability of these faces being returned may be different from the monetary subjective probability, we considered FACE2 in our full model with different distortion indices ($\lambda_{s}$) for the subjective probability as shown in Eq 8.


$$FACE2=\beta_{win}*Wp^{\lambda_{1}}-\beta_{nowin}*{\left(1-Wp\right)}^{\lambda_{2}}+\beta_{fu}*entropy$$ (8)

As stated above, the third term of Eqs 7 and 8 denotes the feedback uncertainty of returned facial expressions. By allowing either a positive or negative sign for $\beta_{fu}$, we can express both gambling-promoting and gambling-inhibiting effects of facial expression feedback uncertainty.

We included the effect of the previous choice and outcome in Eq 9, where $\beta_{pre}$ is the coefficients for the previous choice and outcome. When the previous choice was “gamble,” Choice and Outcome_*t*-1_ takes a value of 1 if the outcome is win, and a value of −1 if the outcome is nowin. Choice and Outcome_*t*-1_ is 0 if the previous choice is “safe.”


$$previousResult_{t}=\beta_{pre}*Choice\&Outcome_{t-1}$$ (9)

The utility functions for gambling and safe options in the full model are summarized by Eqs 10 and 11.


$$U_{gamble}=MONEY+FACE2+previousResult$$ (10)

$$U_{safe}=\beta_{safe}*R_{s}^{\theta_{3}}$$ (11)

where $\beta_{safe}$ and $\theta_{3}$, respectively, represent the coefficients for monetary safe reward and subjective distortion index of the monetary reward in the safe options in the gain frame.

The probability that the participant chose the gambling option is given by the softmax function


Pgamble=11+e−Λ0Ugamble−Usafe (12)

where $\Lambda_{0}$ is the amount of “randomness” in the participant’s choices.

Best-fitting parameters were estimated using a maximum likelihood estimation procedure: $\Lambda_{0}$ was estimated across all trials, and other parameters were estimated separately for each observer condition. We compared models by removing terms one by one using the AIC and BIC scores.

Since any parameter in the computational models can be estimated either commonly or separately for avatar and human conditions, the number of possible combinations is massive. Therefore, we adopted a two-stage approach for the model selection. In stage 1 (S1 Table), we determined the forms of the best models by assuming that all parameters in each model are separate for the avatar and human conditions. Each term in the full model was evaluated by removing a term one by one, and the best models were selected by AIC and BIC. The best models in stage 1 comprised the feedback uncertainty term (FU in S1 Table) and the subjective reward and subjective safe reward terms (S1 Table, #16, Eqs 13 and 11 [AIC]) and the subjective reward and subjective safe reward terms (S1 Table, #7, Eqs 14 and 11 [BIC]).


$$U_{gamble}=subRew_{gamble}+\beta_{fu}*entropy$$ (13)

$$U_{gamble}=subRew_{gamble}$$ (14)

In stage 2, we determined the optimal model (based on the two best models in stage 1, i.e., #16 and #7 in S1 Table) by deciding which parameters should be estimated separately for the avatar and human conditions (S2 Table). This model selection was conducted based on the sum of AIC and BIC using both behavioral and fMRI samples.

The best model consisted of the subjective expected reward gambling, the subjective safe-option reward and the valuation of feedback uncertainty (i.e., entropy), and only the coefficients for the feedback uncertainty term were estimated separately for the avatar $\left(\beta_{fu}^{A}\right)$ and human $\left(\beta_{fu}^{H}\right)$ conditions (#16−6 in S2 Table, Eq 13). This finding suggests that individual differences in valuation of feedback uncertainty affected differential gambling behaviors between the avatar and human conditions.

The effects of subjective probability were also examined, since subjective probability alters in patients with gambling disorders [36]. To determine whether subjective probabilities should be included in the best model, we compared the sum of AIC and BIC of our best model and the model in which subjective probability was replaced with objective probability (S1 Table, #10). The alternate model resulted in a sum larger than that of the best model. We also found that the subjective probability parameter should be commonly estimated for both avatar and human conditions. These results suggest that the effect of subjective probability on the differential gambling rate in human and avatar conditions is smaller than that of the valuation of feedback uncertainty.

### fMRI analysis

#### fMRI image acquisition.

MRI scans were performed on a Siemens 3T Prisma scanner with a 64-channel coil at NICT CiNet using an echoplanar imaging (EPI) sequence with the following parameters: repetition time (TR) = 2,000 ms, echo time (TE) = 30 ms, field of view (FOV) = 200 mm, slice thickness = 2 mm, and ascending interleaved slice acquisition of 72 axial slices. High-resolution T1-weighted anatomical scans were acquired using the MPRAGE pulse sequence (TR = 1,900 ms, TE = 3.37 ms, FOV = 256 mm, and slice thickness = 1 mm).

#### fMRI data preprocessing.

SPM12 (http://www.fil.ion.ucl.ac.uk/spm) was used for MRI data preprocessing and analysis. Preprocessing included motion correction, co-registration to the participant’s T1 anatomical image, and spatial normalization to the standard MNI T1w template with a resampled voxel size of 2 mm. Co-registered EPI data were normalized using an anatomical normalization parameter. Spatial smoothing was performed using a 6-mm Gaussian kernel. Serial autocorrelation was modeled as a first-order autoregressive model, and the data were high-pass filtered at a cutoff of 128 s. Two participants with severe signal drops in the amygdala were excluded from the image analysis.

### GLM analysis

For each participant, the preprocessed fMRI data were analyzed using an event-related GLM design for a multivariate analysis, where six head-motion parameters estimated during the realignment procedure were included in the regressors of no interest to account for motion-related artifacts during the task. Since we are interested in the differential responses between the avatar and human conditions when the task options are presented, the GLM included the following basic regressors: avatar face presentation, human face presentation, option presentation in the avatar condition, option presentation in the human condition, button press, and feedback. The choices (gamble or safe) were separated as parametric modulators (1 or −1) at the time of the button press. To find the feedback uncertainty correlated activity, we used the following parametric modulators at the time of each option presentation: the feedback uncertainty (i.e., entropy) of the gambling option, and the expected reward of gambling and safe options. In addition, we included the reward size modulator at the feedback time. We modeled serial autocorrelation as a first-order autoregressive model.

For each participant, the contrast images were constructed for the regressors of interest, for example, the option presentations in the avatar and human conditions. These individual contrast images were utilized in the second-level random-effects group analysis. Since we are interested in “avatar-human” in this paper, we often refer to “avatar-human” as “difference”. In Fig 5a, we aimed to find the “difference” at the option presentation time; the first-level contrast was “the option presentation of avatar condition – the option presentation of human condition”, and the second-level analysis was a one-sample *t* test without covariates. Negative activation in the amygdala indicated lower amygdala activity in the avatar condition.

Next, in Figs 5b, 7a, and 7d, we attempted to find the regions where the activity in response to feedback uncertainty was lower in individuals who were more likely to take risks in the avatar conditions; the first-level contrast was defined as (feedback uncertainty at option presentation time of the human condition − feedback uncertainty at option presentation time of the avatar condition), and the second-level analysis was a one-sample *t* test with differential coefficients of feedback uncertainty ($\beta_{fu}^{A}-\beta_{fu}^{H}$) as a covariate.

Finally, to correct for multiple comparisons, we first used the FWE correction across the whole brain at *p* < 0.05. After identifying the amygdala as our region of interest (ROI), we applied a voxel-wise threshold *p *< 0.001 and a cluster extent threshold of *k *> 100, followed by  small-volume correction (SVC) for ROI analysis. For whole-brain analysis to identify other regions, we applied the same threshold (*p *< 0.001, *k *> 100) and identified the vACC and vSTR, whose activity remained significant even after cluster-level FWE correction at *p* < 0.05. 

### Regions of interests

By comparing brain activity at the option presentation between avatar and human condition, we found the amygdala was the key region (Fig 5a). We used the amygdala ROI of the anatomy toolbox (combined both sides of amygdala) in the analysis of the differential feedback uncertainty activity (Fig 5b). The differential feedback uncertainty activity showed that the response of the CM was correlated with the differential feedback uncertainty sensitivity ($\beta_{fu}^{A}-\beta_{fu}^{H}$), as we expected.

To avoid double dipping, we used the beta values extracted from the center voxel in the predefined anatomical ROIs to examine the correlations between differences in the feedback uncertainty sensitivity and feedback uncertainty-correlated activities. We decided to use the left CM of the anatomy toolbox as the target ROI, according to the results in Fig 5b.

For the vACC, we used the left Cingulum_s32 in the anatomy toolbox based on the result in Fig 7d, and for the vSTR, we used the open meta-analysis tool Neurosynth (https://neurosynth.org) with the term “risk taking” to produce our vSTR ROI (we used the neurosynth as the anatomy tool box does not include vSTR). The MNI coordinates of the center of the amygdala, vACC, and vSTR ROIs were [−22, −6, −14], [−6, 38, −10], and [−12, 12, −6], respecitvely.

### Statistics for correlations

The correlations between the difference in the coefficients of uncertainty and personality scores or gambling rates were examined by linear regressions using the MATLAB function robustfit.

## Supporting information

S1 Table Model selection in stage 1. We constructed models by removing the parameters one by one from the full model and evaluated each of them based on the sum of AIC and BIC. The table displays a list of removed parameters from the full model and the sum of AIC and BIC across participants. The best models were #16 according to AIC and #7 according to BIC.
(XLSX)

S2 TableModel selections in stage 2.Using the models that passed stage 1, we identified which model parameters should be estimated separately for avatar and human conditions based on the sum of AIC and BIC across all participants. The best model was #16−6, which is represented in Eqs 11 and 13. See Materials and Methods, in which only $\beta_{fu}$ is estimated separately in the avatar and human conditions.(XLSX)

S1 Data Gambling rates and coefficients of feedback uncertainty for the sample of behavioral experiments. (a) Gambling rates in the avatar and human conditions are shown. (b) Coefficients of feedback uncertainty in avatar and human conditions are shown.
(XLSX)

S2 Data Gambling rates and coefficients of feedback uncertainty for the sample of fMRI experiments. (a) Gambling rates in the avatar and human conditions are shown. (b) Coefficients of feedback uncertainty in avatar and human conditions were shown. (c) Personality scores (IRI, STAI, LSAS) were shown. One participant (ID37) failed to answer the personality questionnaire. (d) Feedback uncertainty correlated activity in amygdala, vSTR and vACC. Due to the problem of image quality, two participants (ID40 and ID41) were excluded from the fMRI analysis.
(XLSX)

S3 Data Gambling rates for the sample of our pilot experiment. No observer, avatar observer, and human observer conditions were included.
(XLSX)

S1 FigThe differential $\beta_{fu}$ was positively distributed.The density distribution of the differential $\beta_{fu}.$ Open circles on the x-axis represent individual data points. The difference between $\beta_{fu}^{A}$ and $\beta_{fu}^{H}$ was significantly positive (*p* = 0.0021, *t* = 2.96), and the skewness of the distribution was positive (0.379). Numerical data are provided in S1 Data (b) and S2 Data (b).(EPS)

S2 Fig Results of a pilot study comparing gambling rates of three observer conditions: avatar, human, and no-observer (*n* = 11). To examine the possibility that the avatar condition only made the participants unaware of the partner, the inter-individual averages of the gambling rates (top) and the inter-individual averages of the intra-individual gambling rate differences (bottom) were plotted. The pilot experiment consisted of two conditions: the first was the no observer condition (black), and the second condition alternated avatar (red) and human (blue) observers. Consistent with the main results, the difference in gambling rates between the avatar and human conditions was seen within the middle range of the expected value difference indicated by yellow shaded area (*p* = 0.017, red bar). By contrast, we did not find such a difference between human and no observer conditions (*p* = 0.71, white bar with blue error bar). When comparing the avatar and no observer conditions, the gambling rate was significantly higher in the avatar condition (*p* = 0.037, white bar with red error bar). These results suggest that increased gambling rates in the avatar condition are not attributable to the ignorance of observers. Numerical data are provided in S3 Data.
(EPS)

## Abbreviations

AIC: Akaike’s information criterion
ANOVA: analysis of variance
BIC: Bayesian information criterion
CM: central amygdala
EC: empathetic concern
EPI: echoplanar imaging
FA: fantasy
FEW: familywise error
fMRI: functional magnetic resonance imaging
FOV: field of view
GLM: general linear model
IRI: Interpersonal Reactivity Index
LSAS: Liebowitz Social Anxiety Scale
MNI: Montreal Neurologic Institute
PD: personal distress
PT: perspective taking
ROI: region of interest
STAI: State-Trait Anxiety Inventory
TE: echo time
TR: repetition time
vACC: ventral anterior cingulate cortex
VR: virtual-reality
vSTR: ventral striatum
