# Supplementary material for: Feedback from an avatar facilitates risk-taking by modulating the amygdala response to feedback uncertainty
Source: PLoS Biol. 2025 Apr 22;23(4):e3003122. doi: 10.1371/journal.pbio.3003122 (PMC12015647; doi:10.1371/journal.pbio.3003122)
Supplement: Attachment — Submitted filename: ResponseToReviewers.docx [file pbio.3003122.s008.docx]

**Response to editor**

We thank the editor and three reviewers for the constructive suggestions and criticisms, which have significantly increased the importance and precision of our results. In response, we re-analyzed the data and addressed all comments as explained below.

***Comments***
 * Both Reviewer 1 and Reviewer 2 emphasize the need for a clearer statement of the hypotheses under investigation.

***Response***

We hypothesized that variations in sensitivity to partner feedback lead to changes in risk-taking behavior when feedback is presented through an avatar. We explicitly state this in the revised introduction (page 5, line 86).

Additionally, in the Model selection section, we state: “We hypothesized that sensitivity to feedback uncertainty for facial-expression feedback influences the differential gambling rate in the avatar and human conditions” (page 12, line 179).

***Comments***

* Both Reviewer 1 and Reviewer 3 emphasize the need for clarity about
the way in which entropy and uncertainty terms are used

***Response***

We used the term “feedback uncertainty” to refer to entropy throughout the manuscript. In addition, we incorporated “variance uncertainty” to represent the uncertainty in reward in our updated (model-based) analysis, as suggested by reviewer #1. We found that the results for “feedback uncertainty” remained unchanged.

***Comments***
 * Both Reviewer 2 and Reviewer 3 highlight the need for careful
consideration of the orthogonality or otherwise of repeated tests of
activity in the same brain area

***Response***

We thank the reviewers for the comment. To avoid double dipping in the ROI analysis, we used independent anatomical ROIs for the amygdala and ventral ACC; specifically, the left central amygdala (CM) and ventral ACC (the left Cingulum_s32) from the Anatomy Toolbox (page 17, line 252; page 52, line 789 & line 792). For the ventral striatum, we used the open meta-analysis tool Neurosynth (https://neurosynth.org) with the term ‘risk-taking’ to generate our vSTR ROI (page 53, line 793).

***Comments***

* Greater care is needed in the reporting of statistical effects as
requested by R2

***Response***

In the revised manuscript, we used independent anatomical ROIs to avoid double dipping and reported effect sizes along with p values in the statistical results.

***Comments***
 * Further consideration or justification of the PPI results is needed
given both R1’s and R2’s concerns about the interpretability of this
result.

***Response***

We agree with the reviewers that the PPI results in the original submission did not provide meaningful insights. Therefore, we removed the PPI analysis from the revised manuscript.

**Response to reviewer #1**

***Comments***
> Major points
> 1)    It is unclear what the findings really show. One interpretation
seems to be that the weaker the activity of the amygdala, the more
likely people are to take risks and this relation holds in both the
avatar and the human condition. Thus, there may be nothing particular to
the avatar condition, which would be fine but deserve clarifying.
Alternatively direct statistical comparisons indicating avatar
specificity should be made clearer (e.g., illustrating the brain
findings of current Figure 5b with bar plots of the extracted data may
help).

***Response***

We are highly grateful to the reviewer for the insightful comment. Based on the suggestion, we reanalyzed the data and found that a more negative response in the amygdala to feedback uncertainty is associated with a higher probability of taking risks regardless of whether participants chose more gambling options in the avatar condition or the human condition. Therefore, we revised the manuscript as follows (page 20, line 292; Figure 5d).

[ Next, to further investigate the link between the central amygdala response to feedback uncertainty and risk-taking behavior, we divided participants into two groups: risk-takers against avatars (RT^A^; Fig. 5d left panel), i.e., those with higher values of $\beta_{fu}$ in the avatar condition${( \beta}_{fu}^{A}>\beta_{fu}^{H});$and risk-takers against humans (RT^H^; right panel), i.e. those with lower values of $\beta_{fu}$ in the avatar condition $(\beta_{fu}^{H}>\beta_{fu}^{A})$. We compared central amygdala responses to feedback uncertainty with responses to zero separately for the avatar and human conditions within each group and found that for RT^A^, the central amygdala response was negative in the avatar condition (p = 4.7 x 10^-2^, t = -1.73), while for RT^H^, the central amygdala was negative in the human condition (p = 1.1 x 10^-3^, t = -3.79). These observations indicate that a negative amygdala response to feedback uncertainty drives risk-taking behavior.]

***Comments***
> 2)    The definition of uncertainty is unclear. Is entropy meant to be
the same thing as uncertainty? If yes, why use the term “entropy” at all
(at least in the Shannon version, entropy is independent of magnitude.
By contrast, variance risk, a standard definition of uncertainty, would
be dependent on it). A clear definition and unified terminology
throughout (also in the formulas) are needed. Moreover, the empirical
correlation within the task between the used term (e.g., variance risk)
and other uncertainty terms, including probability should be specified.

***Response***

We incorporated both variance risk for rewards and feedback uncertainty (entropy) in our updated utility function and maintained consistent terminology throughout the manuscript. Using our model selection, we found not only that variance risk was not included in the best models, but also that the results for feedback uncertainty did not change. We explain these changes and results in the model selection section (page 12, line 183) and the computational model (page 43, line 628) section.

***Comments***
> 3)    The computational modeling looks extensive but adds little. First,
with only three levels of probability, modeling probability distortion
does not seem to make much sense. Moreover, it is unclear why loss
aversion is described (it does not feature in the tables and there were
no losses, such that it would make sense to limit the modeling to the
gain domain).

***Response***

We agree with the reviewer that nonlinear probability distortion does not play a major role in the present task setting. However, we considered a possible small effect at the individual level. Therefore, we included both linear and non-linear probability models in the model selection stage and compared them. We included the loss aversion term in our full model since some participants told us in the post-experimental interview that they felt as if gambling would decrease the reward amount that they were supposed to receive (page 43, line 626). Again, we relied on the model selection to determine the optimal terms for explaining the data.

***Comments***
> 4)    Compared to the modeling, a more important question would be
whether the outcome in the current trial explains the choice in the next
trial. In principle, the authors expect such a relation but do not
really show it.

***Response***

We incorporated the effect of the previous outcome and reanalyzed the data. We found that this term was not included in the best models. We explain this result in the model selection section (page 12, line 184).

***Comments***

> 5)    The gPPI analysis does not seem to add much, given that it is
probably driven by co-activation of the amygdala and the striatum.
Unless the authors can convincingly show that this is not the case, this
analysis could be removed.

***Response***

We agree with the reviewer that the PPI results in the original submission did not provide meaningful insights. Therefore, we removed the PPI analysis from the revised manuscript.

***Comments***
> 6)    It is often unclear what contrast exactly was analyzed for the
imaging data (e.g., onset regressors vs. parametric modulators;
comparing what to what). In each figure and each result description this
should be clarified.

***Response***

As suggested by the reviewer, we clarified the contrasts that we used in the individual analysis in the General linear model (GLM) analysis section (page 50, line 747) and the results (page 19, line 281) as well as in the legend of Figure 3.

***Comments***
> 7)    The authors should plot not only the data of the group who showed
more gambling in the avatar condition but also the data of the remaining
group, both in Figures 6 and 8.

***Response***

We revised Figures 5d, 7c and 7f as suggested by the reviewer.

***Comments***

> Minor
> 1)    Figure 2a,b: It is counterintuitive that the overall gambling rate
increases with the difference in expected value of safe-gamble. If
anything, it should decrease. There may be mislabeling of the x-axis (i.
e., it should read “gamble-safe” instead of “safe-gamble”)?

***Response***

The reviewer is correct. We corrected the labeling.

***Comments***

> 2)    Power calculation: what previous research are the medium and small
effect sizes based on? This needs to be specified.

***Response***

According to Lipsey & Wilson (1993), the effect size in psychological experiments is generally of medium magnitude on average. Therefore, we assumed the medium effect size based on Cohen’s d (Cohen, 1988) for the behavioral data analysis.

For amygdala imaging, we initially used an effect size of f^2^=0.15, which corresponds to a medium effect size in the linear regression. However, there was an error in our description, in that we mistakenly labeled the effect size as small. To verify our assumption, we calculated the actual effect size from our previous study on amygdala imaging in response to inequity, finding it was slightly larger than 0.15. Therefore, we concluded that our original assumption was appropriate. We explain in the revised participants section (page 37, line 512).

Lipsey, M. W., & Wilson, D. B. (1993). The efficacy of psychological, educational, and behavioral treatment: Confirmation from meta-analysis. *American Psychologist, 48*(12), 1181–1209. [https://doi.org/10.1037/0003-066X.48.12.1181](https://psycnet.apa.org/doi/10.1037/0003-066X.48.12.1181)

Cohen, J. (1988). Statistical Power Analysis for the Behavioral Sciences (2nd ed.). Routledge. https://doi.org/10.4324/9780203771587

***Comments***
> 3)    At the behavioral level, was there any evidence for a non-linearity
in the relation of the avatar to the human face, as a function of
perceived similarity, and if so, was that reflected in risk taking?>

***Response***

Unfortunately, we did not take a questionnaire about the similarity among avatars and humans and therefore cannot address the issue.

***Comments***
> 4)    The legend to Figure S3 appears to be cut off.

***Response***
We removed Figure S3 from the revised manuscript.

**Response to reviewer #2**

***Comments***
> Major comments:
> 1.    What was the context in which participants were making selections
during the task? What did making an action have to do with the feedback?
Was it just the participants were motivated to receive positive feedback?
 I am having trouble understanding why the participants would care about
the valence of the feedback if they were explicitly told that their
choice had no consequence. Why would I as a participant want to make the
”risky” choice? Were participants told there was a potential to earn
more money in the risky choice (perhaps I missed this point).

In the method section,

[Participants were asked to choose one of the two options by pressing a button within 3 seconds. If the participant chose the safe option, he/she obtained the reward, and the task proceeded instantly to the next trial. If the probabilistic choice was selected, the outcome depended on the win probability presented in the option. The participants were explicitly informed that their choices had no effect on the observer's gratuities. We confirmed after the experiment the recognition of the independence of the observer's reward and their own reward. All they had to do was to earn more of their own rewards.]

***Response***

We agree with the reviewer that clarifying the participant’s motivation and mindset is very important and apologize for the misleading descriptions in the original manuscript.

In the instruction phase of the task, both the participant and the observer were informed that the observer would evaluate multiple dimensions of the participant’s decision-making by filling out a predefined form. They were also told that the observer would receive more detailed instructions later and that the evaluation results would not be revealed to the participant. (page 39, line 545).

Notably, although the participants were informed that the observer evaluated risk-taking behaviors across multiple dimensions and completed a predefined form, they were not given any specific guidance regarding the role of the observer’s facial expressions in the task. The participants were also informed that their choices had no effect on the observer's gratuities and that the observer’s evaluation had no effect on their rewards. We hypothesized that participants change their risk-taking behavior in the avatar and human conditions as an effect of emotion. After the experiment, we confirmed that participants understood their own rewards and the observer's rewards were independent and that they believed they were connected with their partner online via cameras. (page 40, line 563).

Our fMRI results, which suggest that emotional signals from the amygdala influence standard risk-taking decision-making processes in the vSTR and vACC in a bottom-up manner (i.e., from emotion to cognition), are consistent with our view above. We have incorporated this interpretation into the revised discussion section (page 27, line 403).

***Comments***
> 2.    Figure 1 description states that the participants were told that
the observer would evaluate the participant’s gambling behavior,
contradicting what was said in the methods section about there being no
explicit information given to the participants about their choices
resulting in different feedback by the observer.

***Response***

We apologize for the lack of clarity in our original task description. We revised the descriptions in the task section as follows (page 40, line 563).

[Notably, although the participants were informed that the observer evaluated risk-taking behaviors across multiple dimensions and completed a predefined form, they were not given any specific guidance regarding the role of the observer’s facial expressions in the task. The participants were also informed that their choices had no effect on the observer's gratuities and that the observer’s evaluation had no effect on their rewards. After the experiment, we confirmed that participants understood their own rewards and the observer's rewards were independent.]

***Comments***
> 3.    Statistics reporting
> a.    The MANOVA results on page 21 are confusing as the authors state “
The main effects of the reward for the safe option and the expected
value difference were significant” and then list “p = 0.23” for the
observer’s appearance analysis. b.    This is also done on page 23 for the fMRI behavior results.

***Response***

We revised the ANOVA results to focus on significant factors.

>> c.    No correlation values (effect size) reported for correlations on
page 29.

***Response***

We added the effect size for all correlation results.

***Comments***
> d.    Page 39, the amygdala and ventral striatum activity correlations to
the PT and EC scores need effective sizes report. Although these seem to
be biased by double dipping (see major comment 4).

***Response***

We added the effect sizes to the legend of Figure 6 and removed the PT and EC results for the ventral striatum in the revised manuscript. We addressed the reviewer’s concern about double dipping by using anatomically defined independent ROI, as suggested. We explain the anatomical ROIs in more detail in our responses to the following comments.

***Comments***
> 4.    The fMRI analyses seem to be biased by double dipping, particularly
in the sections on pages 30-36. In my understanding of the paper, the
authors functionally defined ROIs for the amygdala and ventral striatum
using a contrast from the task of avatar vs human conditions. They then
used these ROIs to relate the activity to behavior metrics and
parameters from their computational modeling. In my opinion, this
suffers from a double dipping bias because the ROI voxels are selected
based on their sensitivity to avatars vs humans, and then correlated
with metrics related to avatars vs humans, inflating these correlations.
The authors should instead use anatomically defined ROIs, such as from
an atlas, instead of relying on whole-brain analyses to create ROIs.
This would separate selection and testing. I believe the authors used an
amygdala ROI from an atlas, but then combined it with their functionally
defined ROI. Perhaps the authors can solely rely on that atlas for
defining the amygdala and ventral striatum.
> a.    Kriegeskorte, N., Simmons, W., Bellgowan, P. et al. Circular
analysis in systems neuroscience: the dangers of double dipping. Nat
Neurosci 12, 535-540 (2009). <https://doi.org/10.1038/nn.2303>

***Response***

We are highly grateful to the reviewer for pointing out this critical issue. As suggested by the reviewer, we used independently defined anatomical ROIs for the central amygdala and ventral ACC; specifically, the left central amygdala (CM) and ventral ACC (the left Cingulum_s32) from the Anatomy Toolbox (page 17, line 252; page 52, line 789 & line 792). For the ventral striatum, we used the open meta-analysis tool Neurosynth (https://neurosynth.org) with the term ‘risk-taking’ to generate our vSTR ROI (page 53, line 793).

***Comments***

> Minor comments:
> 1.    MANOVA was stated in the methods section vs ANOVA in results (page
21).

***Response***

ANOVA is correct. We corrected the description (page 43, line 618).

***Comments***
> 2.    If the authors are report whole-brain results, I suggest they
include a less stringently thresholded map to be more transparent.
Perhaps there are other regions that are also important and worth
considering. Something in the spirit of Taylor et al., 2023, although I
do not expect the authors to completely redo their analysis in AFNI to
present the same style maps from that paper. If the authors were to rely
on anatomically defined ROIs, as suggested in major comment 4, the whole
-brain analysis could be included as a more exploratory analysis,
revealing other regions that might be involved. Or the whole brain
analysis can just be skipped since the authors have good hypothesis for
examine the amygdala and ventral striatum.
> a.    Taylor, P. A., Reynolds, R. C., Calhoun, V., Gonzalez-Castillo, J.,
Handwerker, D. A., Bandettini, P. A., Mejia, A. F., & Chen, G. (2023).
Highlight results, don’t hide them: Enhance interpretation, reduce
biases and improve reproducibility. NeuroImage, 274, 120138.
<https://doi.org/10.1016/j.neuroimage.2023.120138>

***Response***

As suggested by the reviewer, we conducted a whole-brain analysis where first-level results for correlations with differential feedback uncertainty were weighted in the second-level analysis by differential behavioral sensitivity to feedback uncertainty. We used a moderate statistical threshold with the statistical threshold p < 0.001 uncorrected and cluster size > 30. We found significant activity in the vSTR and ventral ACC. Interestingly, these two areas showed robust activity with a cluster level FWE-corrected p-value of 0.000015 and 0.030, respectively. We included these results in the “Other brain regions encoding differential sensitivity to feedback uncertainty” section (page 22, line 328) and Figure 7. In addition, we used independently defined ROIs for the analysis, as explained above.

***Comments***
> 3.    I believe the authors can motivate their readers more about the
importance of their study. In the introduction, there did not seem to be
much motivation for why the authors would expect there to be differences
between participant behavior to avatars versus humans, and why such a
difference might be important to examine. In the discussion, I believe
the authors could speculate more on how could using avatars versus “real
” people bias a study and results on decision making tasks or other
social tasks.

***Response***
In the introduction, after describing the current limited understanding of how partner avatars affect behavior, we explained not only that companies have begun leveraging avatar-based commercial communication, but also that behavioral and neural changes in risk-taking behavior is important. The corresponding changes to the manuscript are as follows (page 4, line 67).

[Meanwhile, companies have begun leveraging avatar-based communication in commercial settings, particularly in marketing and customer service (e.g., <https://avita.co.jp/avacom/case/7C0vr9m3>, https://jp.cyberlink.com/faceme/insights/cases/302/ntt-data-unstaffed-store-faceme-facial-recognition). For example, on the AVACOM insurance selection platform (https://avita.co.jp/avacom/case/7C0vr9m3), customers can choose to interact with either an avatar or real human when inquiring about insurance products. Reports from companies suggest that avatar use has doubled the number of consultation appointments, possibly because customers feel more comfortable asking questions without concerns about feedback from a real human.

Given the gap between the current scientific understanding of avatars and growing commercial applications of avatar-based communication, there is a need for a systematic investigation of how an interaction partner’s avatar affects behavior and brain function, particularly in risk-taking scenarios. To address this, we designed a task to examine how risk-taking behaviors and underlying neural computations change when a human communication partner's appearance alternates between an avatar and a real person.]

In addition to those changes, in the discussion section, we speculated on how the future use of avatars versus “real” people may bias our communication and social decision making by remarking on “the pros and cons of using avatars” (page 27, line 407).

**Response to reviewer #3**

***Comments***

> Reviewer #3: The study investigates the role of avatar feedback in
modulating risk-taking behaviour and its neural underpinnings.
Conducting both behavioural and fMRI experiments, the research shows
that receiving feedback from avatars, compared to real human observers,
increases gambling behaviour in participants. This increase in risk-
taking is attributed to reduced sensitivity to feedback uncertainty,
associated with changes within the amygdala and ventral striatum.
Participants with higher cognitive and emotional empathy, as measured by
perspective-taking and empathetic concern, exhibited greater differences
in gambling behaviours and neural responses when interacting with
avatars versus human observers. The data suggest that avatars may reduce
anxiety about uncertainty, thereby encouraging riskier choices.
>
> I very much enjoyed reading this manuscript. The task is very
straightforward, and the results are, in my opinion, overall convincing.
I appreciate the replication of the behavioural findings in the fMRI
study. Furthermore, while the modelling is very simple, it helps to
provide an algorithmic interpretation of the findings. Finally, the
statistical analyses are robust. I have only a handful of minor
suggestions that I hope the authors will find helpful during the
revision of their manuscript.

***Response***

We appreciate the reviewer's positive evaluation of our work.

> * I am wondering if the tables for the model comparison can be moved
to the supplemental materials to improve the readability of the results
section.

***Response***
We moved the two model comparison tables to the supplementary materials, which we believe improved the readability of the results section.

***Comments***
> * The signal in the amygd

ala for the contrast avatar vs human seems
very strong. Maybe it is possible to use this main effect to define, at
subject-by-subject level, an ROI for the follow-up analyses (I believe
that the basic contrast would be orthogonal) instead of the anatomical
ROIs. In this way, the author could have a more precise (subject-by-
subject) mask. This is just a suggestion that authors can feel free to
ignore, since the method they used is also standard.

***Response***

We tried to extract individual ROIs but could not for all participants since individual differences in amygdala activity were huge.

***Comments***
> * Both the description of the results in figure 6 (and the figure
itself) is opaque. I would suggest the authors make them clearer.

***Response***
We completely revised the figure, now designated as Figure 5, to better illustrate that differential sensitivity to feedback uncertainty is encoded in the amygdala. Figure 5 also shows that a more negative amygdala response to feedback uncertainty is associated with a higher probability of taking risks, regardless of whether participants chose more gambling options in the avatar or human condition. We believe the updated descriptions (page 19, line 285) and Figure 5 convey the main result more clearly.

***Comments***
> * In relation to the previous point, I am unsure that the use of the
word entropy to describe ‘feedback uncertainty’ is the best term to
describe it. Entropy is a loaded term, and it risks creating more
confusion. I would suggest using the more explicit term of ‘feedback
uncertainty.’

***Response***

We used the term “feedback uncertainty” to refer to entropy throughout the revised manuscript.
***Comments***
> * The PPI results seem rather weak, and I am not sure how much they
add to the manuscript. They would be more appropriate in the
supplemental section, perhaps. This would also reduce the number of
figures, which are already quite a few.

***Response***

We agree with the reviewer that the PPI results in the original submission did not provide meaningful insights. Therefore, we removed the PPI analysis from the revised manuscript.

***Comments***
> * The discussion is clear, but it is mostly a summary of the results.
I would advise the authors to expand the implications of their findings
and their overall impact on understanding social interaction in the
context of human-machine interactions that are becoming increasingly
more common in our world.

***Response***

We expanded our discussion on the advantages and drawbacks of using avatars in real-world contexts (page 27, line 407). We also concluded the discussion with the sentence “These findings should provide valuable insights into human communications and social interactions using avatars that are becoming increasingly more common in our world.” (30, line 469).
